# Pathogenicity of the m.1630A > G variant remains elusive if related mutation carriers with similar heteroplasmy rates are asymptomatic

**DOI:** 10.1016/j.ymgmr.2019.100461

**Published:** 2019-02-12

**Authors:** Josef Finsterer

**Affiliations:** Krankenanstalt Rudolfstiftung, Messerli Institute, Veterinary University of Vienna, Vienna, Austria

**Keywords:** Mitochondrial, mtDNA, Phenotype, Genotype, Pain, Myalgia, Cramps, Respiratory chain

Letter to the Editor

With interest we read the article by Uittenbogaard et al. about a 24yo female with MELAS due to the variant m.1630A > G with heteroplasmy rates of 75% (blood), 95% (urine), and 89.6 (fibroblasts) [1]. Her mother was asymptomatic but carried the same variant with similar mutation loads [1]. We have the following concerns.

The asymptomatic mother with heteroplasmy rates similar to those of her symptomatic daughter suggests that the m.1630A > G variant was not pathogenic and that a mutation in another gene may be causative. Since the results of WES do not convincingly explain the phenotypic difference between mother and daughter, whole genome sequencing (WGS) is recommended.

Patients carrying mtDNA variants may be asymptomatic [2]. Thus, the mother should be investigated for asymptomatic involvement of the brain, eyes, ears, endocrinium, myocardium, lungs, intestines, kidneys, bone-marrow, cartilage, and skin.

We do not agree with the statement about a big difference of the similar heteroplasmy rates between proband and mother in urine and fibroblasts. The difference in blood heteroplasmy rates is not striking either. Heteroplasmy rates in blood frequently do not correlate with the severity of the phenotype [3].

The strongest difference between mother and daughter, in addition to the phenotype, is the fact that the daughter, but not the mother, was regularly taking antiepileptic drugs (AEDs) and immunosuppressants. Some AEDs (e.g. phenytoin, valproate, phenobarbital carbamazepine) can be mitochondrion-toxic and strongly influence the condition [4]. There are also reports about immunsuppressive medication deteriorating MELAS [5]. We thus should know which AEDs/immunosuppressants the proband was regularly taking.

Overall, this study could be more meaningful if heteroplasmy rates in affected organs were compared, if the mother was investigated for subclinical involvement, if the effect of the medication on the phenotype was discussed, if the medication of the index case was provided, and if deletions, indels, duplications, insertions, splice-site variants, and repeat-sequences were excluded.

## Conflict of interest

There are no conflicts of interest.

## Funding

No funding was received.

## Author contribution

JF: design, literature search, discussion, first draft, SZ-M: literature search, discussion, critical comments.
